# Fossil Shell Flour in Livestock Production: A Review

**DOI:** 10.3390/ani9030070

**Published:** 2019-02-26

**Authors:** Olusegun O. Ikusika, Conference T. Mpendulo, Titus J. Zindove, Anthony I. Okoh

**Affiliations:** 1 Livestock & Pasture Science Department, Faculty of Science & Agriculture, University of Fort Hare, Alice Campus, Alice 5700, South Africa; tmpendulo@ufh.ac.za; 2 SAMRC Microbial Water Quality Monitoring Centre, University of Fort Hare, Alice Campus, Alice 5700, South Africa; aokoh@ufh.ac.za; 3 Applied Environmental Microbiology Research Group (AEMREG), Biochemistry & Microbiology Department, University of Fort Hare, Alice Campus, Alice 5700, South Africa; 4 Department Animal Science, Faculty of Agriculture & Environmental Science, University of Science Education, Bindura 263, Zimbabwe; zindovetj@gmail.com

**Keywords:** fossil shell flour, parasites control, performance enhancer, mycotoxin control, stored grain pest control, water purifier

## Abstract

**Simple Summary:**

Fossil shell flour or Diatomaceous earth is made up of amorphous silicates with important physical and chemical characteristics that enable it to be used for different purposes including uses in livestock production. The substance is nontoxic, cheap, and readily available in large quantity in many countries. Recently, fossil shell flour has been modified as additives for several uses. Recent studies have supported its use as animal growth promoter, vaccine adjuvant in livestock, water purifier, mycotoxin binder, inert dust applications in stored-pest management, pesticide, animal feed additive, as a natural source of silicon in livestock, and as natural anthelmintic. Numerous advantages of fossil shell flour include its low-cost and availability, its nontoxic characteristics, and the fact that food grade diatomaceous earth is safe for human consumption. Likewise, all farmers, whether commercial, small-scale, or communal, can make use of fossil shell flour. The numerous uses of fossil shell flour give room for all types of farmers to explore the various benefits and applications of fossil shell flour. It is believed that through this publication, the potential of fossil shell flour will be exposed and explore by many countries.

**Abstract:**

Fossil shell flour (FSF), also known as Diatomaceous earth, or diatomite, consists of amorphous silicates with important physical and chemical characteristics, including porosity and permeability, low density and thermal conductivity, tiny particle size, high surface area, solubility, hydrophobia, and absorption capabilities, which are molecular filter actors, substituting their integral cations without physical changes. The substance is nontoxic, cheap, and readily available in large quantity in many countries. Recently, FSF has been modified as additives for several uses. Recent studies have supported its use as animal growth promoter, vaccine adjuvant in livestock, water purifier, mycotoxin binder, inert dust applications in stored-pest management, pesticide, animal feed additive, as a natural source of silicon in livestock and as natural anthelmintic. Numerous advantages of FSF include its low-cost and availability, its nontoxic characteristics, and the fact that food grade diatomaceous earth is safe for human consumption. In this paper, we review the main uses of FSF in the livestock industry, with reference to similar works earlier published that elucidate their important roles.

## 1. Introduction

Increase in the world’s population has placed ongoing demand on agriculture, especially in the livestock sector, for food security [1]. However, livestock farming faces challenges due to increases in the prevalence of gastrointestinal parasites and other disease-causing parasites, as well as low quality feeds which has resulted in compromised feed conversion efficiency and thus growth rates [2]. As a result, farmers use diets supplemented with feed additives in order to promote efficiency. Antibiotic feed additives are commonly used. This chemical-based feed additive is expensive and the residue on animal products has health implications for the consumer. In addition, the persistent use of antibiotics has caused the spread of antibiotic-resistant bacteria to animals and humans. Therefore, there is a need to replace antibiotics with naturally-occurring feed additives such as prebiotics, probiotics, feed enzymes, herbal extracts, and organic acids, in order to have healthier meat and still achieve optimum production [3]. One such alternative is fossil shell flour (FSF).

Fossil shell flour (FSF), or diatomaceous earth, has numerous uses, including in water purification, as a performance enhancer in livestock, as a mycotoxin binder, and in stored grain pest control. In addition, it can be used as a dietary supplement for animals as well as other agricultural applications [4,5], thus contributing to livestock productivity and consequently food security and safety.

According to the authors of a past work [6] diatomaceous earth consists of geologically-deposited fossilized skeletal remains of siliceous marine organisms and freshwater unicellular species, particularly algae and other diatoms. Diatoms can be described as minute, single-celled water organisms. These minute organisms are confined by a glassy crust. This crust is formed from the silicon dioxide in its source water. Diatomaceous earth is differentiated into two types based on source. One originates from the sea and the other from fresh water such as lakes. Diatomaceous earth from fresh water is preferred because it is richer in silicon dioxide. Diatomaceous earth (food grade) for use in livestock must be crushed until a fine flour is formed. It is then referred to as amorphous silica or fossil shell flour. When viewed with a powerful scientific microscope the miniature sharp edges can be seen, but it physically feels like chalk dust. Many of these fossilized sedimentary layers have been in existence for a minimum of twenty million years in the Eocene and Miocene epochs lakes and seas [7]. The physical and chemical properties of fossil shell flour enable it to play a vital role in livestock production. Previous authors [8,9] have stated that the surfaces of diatoms possess many porous nanostructure silica cell walls or frustules, enlarging its surface area and enabling it to be used as a substance carrier. It has been acknowledged as an animal natural health and sustenance product [10,11], and is described as a fine and pale color silica dust which has the ability to absorb liquids with a definite abrasive characteristic obtained after quarrying, crushing and milling [12,13]. Due to its abrasive property, FSF has been effective when used as vector and gastrointestinal parasite control in ruminants and poultry [14,15,16]. In this paper, we carried out a review of the main uses of FSF in the livestock industry and other areas of human endeavors.

## 2. Physical and Chemical Characteristics of FSF

Fossil shell flour (FSF) is fine, soft, lightweight, pale colored, is of biogenetic sources, and is comprised mainly of amorphous silicon (SiO_2_NH_2_O), derived from the skeletons of diatoms. It is abundantly available on the planet earth and has distinctive physical properties, such as porosity (35–65%), permeability (0.1–10 MD), low density and thermal conductivity, tiny particle size [17], and large surface area [18]. The characteristics of the surface of diatomite, such as acidity, solubility, hydrophobicity, ion exchange, and absorption functionalities, are largely controlled due to the presence of water, which is partly and morphologically connected with the crystal structure of the diatomaceous earth, thereby resulting in vigorous hydroxyl groups [19]. Its unique porosity (typically 10–200 μm), minute particle size, extensive surface area, high permeability, poor thermal conducting properties, and chemical inertness makes it of great interest among naturally-occurring materials [20,21]. Table 1 shows the elemental composition of natural FSF and Table 2 [22] compares the chemical composition of fossil shell flour from various locations. Kilpinen and Steenberg [23] reported that FSF from difference sources have difference therapeutic strength against parasites especially in poultry

Natural fossil shell flour can be changed by treating it with hydrochloric acid to purify the silica surface. This helps to significantly mitigate the input of detrimental calcium, iron, aluminum, magnesium, and alkaline rudiments while dissolving little of the silica [21,31,32]. Table 3 shows the chemical characteristics of modified and natural FSF, obtained through X-ray fluorescence spectrometer. The composition reveals that silicon dioxide (SiO_2_) is the major constituent while small amounts of iron oxide (Fe_2_O_3_) and aluminum oxide (Al_2_O_3_) are also present [32,33]. After modification, fossil shell flour can remove substances such as Lead (Pb^2+^), Copper (Cu^2+^), and other heavy metals optimally. One of the elements used in diatomite modification is magnesium oxide, which is achieved by treatment with sodium hydroxide and manganese chloride [34]. 

## 3. Availability and Accessibility

Fossil shell flour (FSF), or diatomite, has been known for decades and several countries are actively involved in mining, milling, and transformation of this compound. The world production of diatomite in 1981 was 1.5 million tons, close to half of which came from North America [34]. Table 4 shows the major world producers of diatomite and the estimated annual quantity produced. The resources of crude diatomite all over the world are sufficient for the anticipatable future and its production worldwide is used in absorbents (9%), fillers (14%), cement (21%), filter aids (55%), other uses (1%), as well as specific pharmaceutical, biomedical, and agriculture uses.

## 4. Types of Farmers Making Use of FSF

There are serious losses to stored grain suffered by communal farmers in sub-Saharan Africa because of insect damage. However, as many of these farmers cannot afford the cost of chemical protectants, they are resorting to using diatomaceous earth to preserve their produce for their household and livestock use [36]. Diatomaceous earth is cheap, readily available, effective against insects, and safe to use.

All farmers, whether commercial, small-scale, or communal, can make use of FSF. The numerous uses of FSF give room for all types of farmers to explore the various benefits and applications of fossil shell flour. Stathers et al. [36] and Mvumi et al. [37], observed in two different studies that small-scale farmers in Tanzania and Zimbabwe were able to preserve their grains for both their animals and their household by applying diatomaceous earth to the grain at the rate of 0.1% (*w*/*w*). Likewise, Badii et al. [38] reported that diatomaceous earth applied at 1.50 or 2.00 g kg^−1^ at 50% RH is a feasible substitute for preventing *C. maculates* infestation in stored Kersting’s groundnut for commercial and small-scale farmers.

Another opportunity that is open to commercial, small-scale, and even communal farmers in the use of diatomaceous earth is that food grade diatomaceous earth can be put in a bowl and placed in the pen or barnyard as a mineral lick for the animal [39]. Farmers of different scales of production can therefore make use of diatomaceous earth for their animals to preserve feed ingredients as well as provide a source of minerals as well as other numerous uses.

## 5. Potentials of Fossil Shell Flour

### 5.1. Potential of FSF in Parasite Control in Livestock

Parasitism in small ruminants is a major problem for farmers. By their nature small ruminants, especially sheep, graze close to their own dung, which exposes them to parasitic ova and subsequently parasite load [40]. Consequently, parasitic gastroenteritis continues to be a health risk and constraint to the production of small stocks due to associated disease, mortality, control measures, and cost of treatment at clinical as well as subclinical levels [41]. Economically, a reduction in profitability levels of ~15%, including weight loss of ~50% on account of intestinal parasites, have been reported [42,43]. This loss is a result of low production on account of poor growth, low weight gains, and poor utilization of feed [44]. Parasites can cause hematological and biochemical turbulences in sheep [44], as well as anorexia, altered water and electrolyte balance, anemia, poor reproductive performance, and weight loss, which can consequently lead to an increase in the mortality rate of lambs [14]. Studies have revealed that GIT parasites are significant causes of production losses in sheep, particularly in sub-Saharan Africa, [14,45]. At any stage in the growth of sheep, they can be vulnerable to gastrointestinal nematodes, although lambs and peri-parturient ewes are most epidemiologically affected [46]. One of the main organisms that economically restricts sheep production worldwide are gastrointestinal nematodes [15]. The most important factor which limits the control of this parasitic organism is the stable increase of anthelmintic resistance globally, particularly if animals are underdosed or treated under preventative and suppressive treatment regimes. Therefore, alternative and/or complementary sustainable control programs would be beneficial [47,48].

For the past two decades’ fossil shell flour (FSF) has been used to naturally deworm animals. The authors of a previous paper [5] clearly demonstrated that a 2% inclusion rate of FSF can be used with positive results in the destruction of internal parasites and worms. In the study, the inclusion of FSF increased the productivity and profit on dairy and beef cattle farms as a viable alternative to synthesized chemical products. The National Experimental Council and the National Council of Organic Standards in the USA discuss the disadvantages of chemical inoculations in their database of published products and suggest that Diatomite be used as an alternative [5]. A previous study [49], on the efficacy of fossil shell flour with ewes and lambs, reports that when feeding sheep with a combination of FSF and a mineral supplement, with no other anthelmintic used, at a ratio of 1:1 for three months, the incidence of Haemonchus in ewes was minimized compared with the control.

Lambs fed with 2% inclusion rate of FSF appeared to have an earlier gain in weight, tails that were cleaner, and shining wool [49]. Also, great improvement in the general body condition of the lambs was reported. Deutschlander [49] also performed a similar study using heifers. He reported that heifers that were five hundred-pound in weight that were fed FSF pasture on free-choice conditions showed no worms either mid- or late-season. The heifers consumed FSF of about one pound per week per heifer and when the dairy cows were not fed FSF for a few days they craved the substance and consumed several pounds as soon as it was made available to them. Despite the heavy feeding on FSF no side effects were recorded in the cows and they remained in good condition. He concluded that the lambs performed much better than the ewes and cattle while on pasture. In addition, the use of FSF recorded savings of $1.50 per head for both ewes and cattle as a dewormer over the use of conventional medicines. Deutschlander [49] observed that the body conditions of the heifer and ewes were very satisfactory when they were withdrawn from pasture. Another study Bernard et al. [50] observed that Spanish/Boer cross goats fed varying inclusion levels of FSF at 1.77 g, 3.54 g, and 5.31 g per kg had significant improvement in mean weight gain and fecal egg count as the inclusion levels increased. Similar observations were reported by Bennett et al., Mclean et al. [4,51]; both observed that FSF significantly increased body weight, feed conversion efficiency, and growth rate, and decreased parasite load of the experimental animals. However, they both postulated that for an effective and optimal outcome, FSF should be fed for a longer time period. They hypothesized that the abrasive edges of the diatom particles injured the cuticles of nematodes when in contact, resulting in dryness and eventually the death of the parasite. In line with these findings, Ahmed et al. [52], considered the use of biological control of gastrointestinal parasites in Merino sheep, feeding fossil shell flour as 2% of their diet. They also reported that fossil shell flour had an efficacy of 61%, although efficacy varied with time. 

As an alternative to anthelmintic, fossil shell flour (FSF) was evaluated by [53] for its ability to inhibit the migration of *Oesaphagostomum dentatum* larvae using migration and inhibition assays in vitro, in unsheathed and sheathed third stage larvae. They observed that FSF was more effective in unsheathed larvae at 0.3 mg/mL after 20 h with 61.6% inhibition. With sheathed larvae, FSF had a significant effect of 1 mg/mL exposed within 24 h with 67.6% inhibition. This shows that the presence of cuticle could reduce the effectiveness of FSF as anthelmintic. There is therefore a need by researchers to investigate parasite cuticle abrasion by using equipment such as scanning electron microscopy to examine the cuticle integrity from treated versus untreated animals. In a separate experiment, Osweiler and Carson, Fernendez et al. [40,54] contrarily observed that FSF did not lower the parasite load in lambs. 

Fossil shell flour has also been reported to reduce ectoparasites. Dawson [55] reported a significant reduction in bird flea population sizes (*Ceratophyllus* ideas) and several blow fly species (*Protocalliphora* spp.) in the same year. Fossil shell flour seemed to be more efficient at reducing the flea populations, perhaps because fleas have body sizes that are smaller and so are more susceptible to dehydration on account of FSF’s abrasive action. It was also confirmed, in more recent researches by Martins and Mullens [56] and Amy [57], that FSF is able to suppress the activities of Northern fowl mites (*Acaris macronyssidae*). Kilpinen and Steenberg [23] reported that FSF, is one the commonly used substitute control methods for poultry red mites (*Dermanyssus gallinae*) in Europe because it kills the target host mainly by desiccation. The authors also observed large differences in the types of FSF versus red mites’ mortality. Therefore, there is needs for other researchers to investigate difference FSF formulations head to head for parasite control 

### 5.2. Potential of Fossil Shell Flour as Detoxifier in Livestock Feed

Animal feeds are often contaminated with many pathogenic microorganisms at different stages of the manufacturing process, despite concerted effort at averting these [10]. Chief among the microorganisms are Aspergillus and Fusarium genera that produce mycotoxins including Ochratoxin A, Aflatoxin B1, Deoxynivalenol, Fumonisin B1, and Zearalenone. Of the 300 to 400 known mycotoxins, deoxynivalenol (DON) and aflatoxin (AF) are known as the most common and most detrimental to animal industries [58]. According to Agag, Jones et al. [59,60] one-quarter of the world’s crops are infected with mycotoxins. The North Carolina Cooperative Extension Service also established that close to one-fifth of tested corn contained levels greater than 20 µg/kg AF, and close to two-thirds of corn contains DON Weaver et al, Agag [10,59]. These mycotoxins are usually seen in the feed chain due to infection of crops by fungi, or use of moulds forage and grains as animal feed condiments [60]. 

Richard [61] reported that mycotoxins are produced on growing plants due to fungal invasion during preharvest, postharvest and during crop transportation and storage, and these have been shown to adversely affect man and animals at low levels, and have a substantial effect on international economies and trade. When consumed, these mycotoxins cause a reduction in the growth of animals, as well as immune and reproductive dysfunction and can damage organs [61,62]. These toxins also cause a pathogenic shift in vital organs such as liver, kidneys, and lymphoid tissues [63]. They have been reported in hepatic lesions in chickens causing such effects as enlargement, paleness, hydropic degeneration and necrosis, periportal fibrosis, and bile duct hyperplasia [64]. In addition, the spread of aflatoxin and its metabolites from feed to products such as liver and eggs as well as animal edible tissues [65] have become a predominant prospective hazard for human health. 

Feed decontamination from aflatoxin is a major requirement in livestock industry. Physical, chemical, and biological methods for decontamination are used. Other approaches such as protective actions, good storage practices (GSP) as well as good agricultural practices (GAP) at both preharvest and postharvest periods, have been adopted to control the harmful mycotoxins in animal feed. As good as these options are in mitigating mycotoxin contamination; the use of best practices might not completely avoid or get rid of mycotoxins in the feed chain [66]. An acceptable detoxification procedure must be cost-effective and capable of eradicating all traces of toxin with no detrimental residues, and should not weaken the nutritional quality of the product [67]. One such procedure is the use of fossil shell flour, which has been reported to be effective against mycotoxins. The authors in their experiment using quail chicks observed that addition of FSF to an Aflatoxin (AF)-containing diet significantly reduced the deleterious effect of AF on food consumption, body weight gain, and feed conversion ratio. Food consumption was reduced by 14% in quail chicks consuming the AF diet without FSF, but by only 6% for quail chicks consuming the AF plus FSF diet. Similarly, overall body weight gain was reduced by 27% in birds consuming the AF diet without FSF, but by only 8% for birds consuming the AF plus FSF diet. Parlat [68], reported that broiler chickens fed a diet containing diatomaceous earth (DE) at 400 and 800 mg/kg, respectively, had significantly greater body weight and less feed conversion ratios and an increase in serum total protein and albumin values than those fed a diet containing T-2 toxin at 0.5 ppm and 1 ppm after 35 days of age. However, DE supplemented chickens were not significantly different in relative organ weights of kidney, liver, bursa of Fabricius, spleen and serum biochemical values of AST, ALT, cholesterol, triglycerides, and creatinine when compared to birds fed with only T-2 toxin. The study showed that the inclusion of DE as an additive in the diet was partly helpful in lessening the harmful T-2 toxin effects in broiler chickens. Furthermore, Shivashaankar et al. [69], reported that supplementation of fossil shell flour at 400 and 800 mg kg^−1^ in an aflatoxin-mixed diet (with 0.5 and 1 ppm of AF kg^−1^, respectively) significantly reduced the deleterious effects of AF on the growth parameters of broiler chickens and the serum biochemical values by bringing about a boost in serum total proteins, albumin, triglycerides and cholesterol levels. The same author also recorded a significant increase in ALT, AST, BUN ALP, and creatinine levels in fossil shell flour supplemented broilers. [70], in another study with in vitro models to evaluate the properties of fossil shell flour and other natural absorbent agents on six mycotoxins, namely, ochratoxin A (OTA), aflatoxin B1 (AFL), zearalenone (ZON), diacetoxyscirpenol (DAS), deoxynivalenol (DON), and T-2 toxin using thin-layer chromatography (TLC), observed that fossil shell flour bound more than 95% of applicator AFL and 66.67% of OTA (only diatomite adsorbed this toxin). Binding of DON has also been observed [62], but only at pH 3.0 of an electrolyte. Its adsorption index varies from 25.00 to 50.00%, while the ZON adsorption index ranges from 12.20 to 37%, and for T-2 toxin the adsorption index ranged from 16.67 to 33.33%. These results appear to suggest that fossil shell flour is an effective absorption agent that can be used in the animal feed industry to decontaminate many mycotoxins in animal feed. 

Fossil shell flour can also be used in the production of some mycotoxin adsorbents, and for diarrhea remediation in ruminants and birds [71]. The use of adsorbents such as fossil shell flour as a feed additive is the most effective, economical and healthy solution for the decontamination of mycotoxins livestock feed. It should be modified in accordance with current demand of farmers and should be widely monitored to ensure its safety and effectiveness [72].

### 5.3. Potential of Fossil Shell Flour as Animal Performance Enhancer

Growth promoters and enhancers are materials that facilitate the growth of farm animals, particularly swine, poultry, and livestock, where the value of such animals to the farmer depends partly on body weight. The market for animal growth promoters and enhancers is large and growing. The most widely used group of growth promoters in animal feed is antibiotics [73]. Other growth promoters include probiotics, prebiotics, essential oils, botanicals, enzymes, organic acids, phytochemicals, vaccines, RNAS, antibodies, bacteriophages, antimicrobials, innate defense molecules, immune enhancers, and combinations thereof [74]. There remains room for the development of new growth enhancers, particularly those that can be made in a cost-effective way. In line with this, Abd El-Tawab et al. [75] conducted a study using spent filter media containing ~35% moisture, 32% diatomaceous earth, 22% organic carbon, and 11% activated carbon, and reported that although their results were not definitive, spent filter media improved daily feed intake, weight gain, and efficiency. The animal feed compositions as described herein may be fed to different animals. It is believed that the use of spent filter media may advantageously increase the growth of the animals on a daily basis compared to identical amounts of conventional animal feed compositions, although this result is believed to be affected by other conditions, Abd El-Tawab et al.; Sarijit et al. [75,76], including the composition of the base feed. In addition, Abd El-Tawabet al. [75] also noted that these animal feed compositions did not adversely affect the lean mass percentage of the animals and did not appear to exhibit any harmful effects to the growth or the health of the animals. Likewise, Adebiyi et al. [25] reported that there was a substantial improvement in average weight gain, feed conversion efficiency and bone development of cockerel fed 6% fossil shell flour inclusion level as feed additives throughout the 16 weeks of the trials. Similarly, fossil shell flour significantly improved intake of feed (7.44%), body weight gain (9.51%), and improved ratio of feed conversion (2.08%), as well as the productive efficiency index (5.48%) in cockerel exposed to aflatoxin B1 [25,77]. Fossil shell flour also improved serum albumin (2.26%) and the action of serum LDH (44.4%) in a previous study [78].

Modirsane et al. [79] supplemented the feed of 252 piglets with silicon dioxide (a major component (79%) of FSF) and found improved feed intake by 4.13% and an improved mean daily gain by 3.26% during the general early period, compared to groups not given silicon dioxide. The outcome led to an increment of 2.2% in the piglets’ weight at the expiration of the postweaning phase (24.52 kg vs. 23.99 kg). They concluded that under their study circumstances, an addition of 0.02% crystalline silicon dioxide (fossil shell flour) to piglet feed increases daily feed intake, growth rate, and piglet weight at the end of the weaning period. This mineral supplement could offer prospective financial gain to pig farmers. 

### 5.4. Potential of Fossil Shell Flour as Water Purifier for Livestock Usage

The provision of poor quality drinking water for livestock in semi-arid and arid regions for several months of the year is very common [80,81]. These supplies originate from streams, canals, small wells, or water holes, which are also used for irrigation [82]. This water is often high in salt and toxic elements such as lead, copper, selenium, mercury, and arsenic [32]. They are hazardous to animal health and may cause physiological upset or render the animal products unsafe or unfit for human consumption, as well as possibly cause the death of the animal [83]. The bulk of heavy metals are identified to be poisonous and cancer-causing agents. Problems emanating from toxicity are aggravated by irrigation of forage with the same potentially toxic water. The plants make use of the salts, thus increasing the level of risk in toxicity to the animal when both feed and water sources surpass hazardous levels. This may also occur with selenium. Sources of these toxins and salts are animal feces, birds, animal carcasses, intensive livestock industry runoff from bare paddocks, sewerage waste, veterinary antibiotics, herbicides, and pesticide residues [32]. Diatomite that has been modified with manganese-oxide is an active and good adsorbent for removing heavy metal ions such as, Pb^2+^, Cu^2+^, Cd^2+^ Ni^2+^, and Hg^2+^ [32]. The negative charges on the modified surface is an attribute that enhances adsorption performance. In addition, the enlargement of the surface area is believed to play a vital role in the overall removal process. Therefore, lower loading of diatomite has a superior performance over higher loading adsorbents [84]. Similarly, Al-Degs et al. [85] reported that heavy metal ions such as Pb, Ni, and Cu were removed from wastewater using manganese-oxide modified Iranian diatomite considered this to be an adsorbent and a filtration substance. Also, organic and inorganic substances can be removed from wastewater and surface water through the absorption of diatomite modified with Mn [86] and Bello et al. [32] reported that modified diatomite has a greater removal capacity for heavy metals from water than unmodified diatomite. Walker and; Weatherley [87] established that the use of diatomaceous earth is a promising technique in the removal of heavy metals from wastewater and surroundings. Hence, FSF can be used to treat water/wastewater for animal use, thereby reducing risk for human consumption. Hence, FSF has significant potentials in mitigating water scarcity and turning waste into wealth. 

### 5.5. Potential of Fossil Shell Flour as Source of Minerals for Livestock

Minerals are naturally-occurring substances generally required in microscopic amounts from less than 1 to 2500 mg per day, depending on the mineral [88]. Just like vitamins and other essential food nutrients, mineral requirements vary with animal species. Large mammals require large amounts of calcium for the building and preservation of bones and standard function of nerves and muscles [89]. Phosphorus is an important constituent of adenosine triphosphate (ATP) and nucleic acid, and is also vital for acid–base balance, and the formation of teeth and bones. Red blood cells cannot function properly without iron in hemoglobin: the oxygen-carrying pigment of red blood cells. Iron is also an important component of the cytochromes that function in cellular respiration. Molybdenum, manganese, copper, zinc, magnesium, selenium, and iron are important cofactors found in the structure of certain enzymes and are crucial in many biochemical pathways. Mammals need iodine to make thyroid hormones. Sodium, potassium, and chlorine are imperative in the upholding of osmotic balance between cells and the interstitial fluid [90]. According to Soetan et al. [91] different microminerals, for example, Se, Mn, Zn, and Cu, are important for the optimal performance of the immune structure and for resilience against pathogens. Cobalt deficiency decreases the resistance of animals against helminth infections [92]. Molybdenum also plays an important role in immunity against endoparasite [93] and can decrease the worm burden in lambs [15,94]. Natural food grade FSF contains 15 macro and microminerals, which are important to animal diets. The inclusion of natural FSF to poultry diets has continuously revealed weight gains in production. This gain could be connected to a combination of factors such as the ability of natural food grade FSF to decrease parasite populations, which promotes a reduction in stress on the animal and improved food assimilation [25,95]. Natural food grade FSF contains a wide range of naturally present chelated minerals such as calcium, magnesium, iron, phosphate, sodium, titanium, and potassium. Based on the final optimal levels, the mineral constituents that it provides may replace a small proportion of the entire mineral premix or complex. However, its ability to increase the incorporation of other minerals and microminerals, (particularly the effects of fossil shell flour on improved general mineralization such as bone), may also be the reason for improved performance [96,97,98]. In a study conducted at the University of California, the importance of fossil shell flour for growth performance and maturity in chickens was established. It was reported that the silicon inclusion group had thicker legs and bigger combs relative to their body size, significantly superior to other groups without silicon supplementation [25,96] also reported that growing cockerels fed 6% inclusion levels of FSF had the highest values for Ca, P, and Ash than the control and other treatments. However, tibiotarsi weight, length, and robusticity index were not affected significantly. In livestock, production and health problems are often associated with calcium deficiency in many species of animal. Addition of natural food grade FSF to diets could, therefore, avert any calcium associated performance or health issues. Minerals present in natural food grade FSF can also help to meet the mineral constituents of lactating animals [88]. Table 5 shows the mineral constituent of food grade FSF, which includes major and trace elements needed by livestock for growth performance.

Fossil shell flour is the most abundant form of organic amorphous silica in the world (79% to 94% of silicon) [99]; it exists in silicon dioxide form, it is bioavailable, and is essential for good bone growth and nutritionally important for preventing certain forms of chronic diseases associated with aging [99,100]. Humans, animals, and plants have an essential need for silicon in order to sustain life, and regrettably, in today’s world, our diets can easily become deficient in silicon. 

### 5.6. Potential of Fossil Shell Flour in Feed Storage

Over one-third of the total grains produced globally are lost annually due to pests during storage [101]. In sub-Saharan Africa farmers suffer significant losses to their products as a result of insect damage. These losses undermine their livelihood, food security, reduction in market returns, and also have an indirect effect on the feeding of animals [36]. This loss often increases the cost of production in livestock industries as a result of an increase in the cost of feeds. To prevent these losses, different traditional strategies have been adopted, including mixing grain with ashes or plant materials, and the use of synthetic chemicals (insecticides). Residual synthetic chemicals are the most frequently used protectants against stored-product pests in stored grain. They are often applied directly to the product and provide protection against stored-grain pests as long as the effect of these insecticidal persists [102]. Nevertheless, current frequently used protectants possess numerous disadvantages because of their toxicity to mammals, and the fact that they leave deposits in the product; in addition, many species of insect are resilient to some current protectants [102]. These shortcomings have made researchers assess the use of different control methods such as insect growth regulators, botanicals, biological control, microbial control, and inert dust. Fossil shell flour (FSF) is the most promising alternative that can replace insecticides successfully. This it does by absorbing the epicuticle lipids and fatty acids, thereby resulting in the dehydration of arthropods. FSF comes into direct contact with the insect bodies and absorbs the waxy cuticle of the outer layer, after which the insect loses water and dies [103,104,105]. FSF needs no specialized kit for application on grains, but one can use similar techniques as that used for insecticides. Sabbour and Abd-El-Aziz [106] reported that FSF is tenacious in its mode of operation, poses little or no pest resistance problems, and leaves no residue, but its efficacy depends on the influences of temperature, provenance, humidity, and the individuality of particular pests and substratum. Sabbour et al. [105] reported that calcium hydroxide modified diatomite (Ca-DE) and those modified with sodium hydroxide (Na-DE) were the most effective antidote against insects. Ca-DE yielded better results and accomplished the highest mortality percentages recorded at 88% and 96% for treatments against *R. dominica* and *B. incarnatus* with 1.0%, respectively. The lowest mortality rate was recorded for Al-DE at a concentration of 0.5% and achieved 21 and 15% mortality for the corresponding species, respectively. In a similar study conducted by the same researchers on the use of FSF in pest management in stored-grain, they reported that the rate of reproduction of tested insects was greatly reduced by both FSF and modified FSF, and egg production was greatly reduced by modified-FSF under stored conditions. The average quantity of eggs laid per female and percentage of adult emergence (F1) of each tested insect were appreciably affected by both natural FSF and modified-FSF in comparison to the control. The same authors found that Nano-FSF strongly reduced the number of eggs laid for *T. confusum* but reduced to a lesser extent for *T. castaneum* (3.8 ± 1.5, 17.8 ± 7.5, 26.6 ± 3.5 eggs/female and (13.8 ± 1.5, 37.8 ± 7.5, 46.6 ± 3.5 eggs/female) after 20, 90, and 120 days of storage interval respectively. The constant effect of nanoparticles showed numerous unusual modes of action such as falling oviposition, mature emergence (F1), and influx percentages of hardened insects. Sabbour et al. [105] concluded that FSF-nanoparticles and natural FSF can be a good instrument in pest management programs for *T. confusum* and *T. castaneum*. Badii et al. [38] in their studies on the prevention of *C. maculates* infestation in stored Kersting’s groundnut, reported that mortality of the adults increased gradually with the increased quantity of FSF and the contact period. Grains that were treated at 2.00 or 1.50 g kg^−1^ documented considerably fewer eggs and a lower rate of F1 emergence compared with dosages with lower quantity. Increased FSF concentration constantly decreased produce weight as a result of low beetle numbers however, a significant difference on seed viability was not recorded. FSF was more efficient at 50% relative humidity than at 80% relative humidity.

It appears that FSF can be used by communal farmers, animal feed mill operators and commercial farmers to preserve grains for their animals during the time of surplus to the time of scarcity. It is cheap, nontoxic, readily available and requires no equipment for administration. FSF likewise has broad uses as an anticaking agent in the storage of grain and in feed mixing. This enables for better flow ability, mixing and handling and prevention of particles from clumping together. According to Bannett et al. [4], food-grade fossil shell flour placed in livestock feed may help discourage the growth of fleas and other dangerous organisms.

### 5.7. Potential of Fossil Shell Flour on Quality of Wool and Mohair

In the face of demand for lighter weight fabrics by consumers and the rigorous challenge of competition from synthetic fabrics, producing quality wool that will translate to the yearning of the final consumer has been of utmost important to the sheep industry in the past 25 years. When looking for fine, uniform, and high-quality wool appropriate for the textile industry, Dohne-merino and other merino sheep come to mind [107]. Wool fiber diameter and fiber length are the key characteristics used to evaluate the processing route and final quality of the finished textile products [8]. Factors such as genotype, nutrition, age, and sex determine the quantity and quality of fleece from sheep. Cilek [108] reported that the fiber diameter and the proportion of kemp are greatly influenced by the genetic composition of the animal. Fiber diameter variability and proportion of medullated fiber are attributed to the sex of the animal. Shah and Khan [107] observed that females have a greater measure of fiber diameter variability and proportion of medullated fiber than males; and that age on all fleece traits was significant. The effect of sex was statistically noticeable for all fleece traits except for fiber tenacity and comfort factor. It can be generally concluded that younger sheep and rams have higher fleece yield and fleece quality than older sheep and ewes. Similarly, the age of the animal and the fiber diameter are directly proportional to each other, while age and fiber staple length are not affected by age in merino sheep. Fiber diameter is known to be affected by the quality and quantity of nutrients available to the animal, and is related to live weight [109,110,111].

Variation in staple length relates positively with live weight. McGregor et al. [112] observed that the highest correlation occurred between staple length and live weight that were measured simultaneously, with a lower correlation when comparing the two parameters at different time. Also fiber diameter variation mostly influences staple strength [82,110,111]. Staple strength and location of a break are greatly influenced by both the minimum fiber diameter and rate of variation along the fiber. However, nutrition, disease, stocking rate, genotype, and the physiological state of the animal all influence the staple strength. Likewise, climate, altitude, genotype, nutrition and soil, relative humidity, pH within the fleece, the level of perspiration, production of sebum by sebaceous glands, and time of shearing are factors that determine the color of wool [113,114,115].

The effect of gastrointestinal parasites and lice on fleece yield and quality have also been studied. Frequent treatment of antihelmintics increases the fleece yield, fiber diameter, staple length, and strength as well as crimp fleece weight [116,117]. *Bovicola ovis* (lice) infestation has an adverse effect on wool characteristics, and this is amplified as the number of lice present and duration of infestation increases. With lice infestation greasy wool changes color and becomes less bright, staple length is reduced and staple strength is negatively affected. Van Burgel et al. [117] also reported that sheep with modest to severe infestation produce less sound wool (1.7 vs. 3.0 kg/head) and more cast wool (0.4 vs. 0.1 kg/head) than sheep with very few lice.

Since fossil shell flour improves body weight gain, reduces intestinal parasites, and in general might be expected to suppress ectoparasites, it follows that inclusion of fossil shell flour to the diets of sheep will increase fleece yield and improve the quality of wool greatly for good market prices as well as the international standard.

### 5.8. Fossil Shell Flour Can Be a Replacement of Antibiotic in Animal Feed

Fossil shell flour might be able to replace antibiotics as a growth promoter [4,75,79], as a gastrointestinal nematodes eradicator [52,53] and as an eliminator of dangerous bacteria in the body system of an animal [118,119].

Most of the chemical constituents of FSF have a strong negative charge. Just like a magnet, the negatively charged elements of FSF attract all positive bodies that are sufficiently tiny to pass through the porous openings of FSF. These strong charges are capable of attracting a great quantity of oppositely charged substances, whether element, bacteria, or viruses. They pass through the digestive tract to remove these dangerous substances from the body system. 

When diatomite is added to the feed of animals, Gram-positive bacteria, which are normally targeted in ruminant animals through the use of antimicrobial feed additives, may also bind to the negatively charged shells. This could promote FSF as an efficient replacement for antimicrobial and antibiotic products commonly used to carry out these roles. James et al. [118] in their study to evaluate the impact that diatomite (2% concentration) has on the adsorption of *Escherichia coli* (ATCC 25922), reported that the highest adsorption capability of 10.99 mg g^−1^ was realized within 26 h with a solution of 12 mg L^−1^ As (III) at pH 4. Another study carried out previously [119] on the adsorption ability of diatomite also demonstrated the superior capacity of diatomite to neutralize bacteria, comprising Gram-positive *Staphylococcus aureus* and Gram-negative *E. coli*.

### 5.9. Other Potentials of Fossil Shell Flour in Feed Industry and Livestock Production

Fossil Shell flour as adjuvants vaccine: Deactivated vaccines are normally used in poultry as part of a complete vaccination procedure. These vaccines are able to stir up antibodies of high titers, capable of protecting against general infections, and which are transmitted from parent stock to their offspring as maternal antibodies. In order to boost their immunogenicity, these vaccines have adjuvants. Common adjuvants often used in poultry vaccines are aluminum hydroxide ointment (Alum) and ASO_4_ (oil-based type) [120,121]. Substances that are added to a vaccine to accelerate or enhance the body’s immune response to the vaccine and to reduce the quantity of antigen needed for vaccines are referred to as adjuvants [122]. According to Waksman and Hunter [123,124] there are three different mechanisms to accomplished this. First, a prolonged immune response is induced by a slow release as a result of the deposit of antigens formed at the injection site. Second, particulate antigens are formed, which antigen-presenting cells can detect. Finally, local inflammation may be caused by adjuvants that initiate system-recognition receptors, activating antigen production. However, undesirable effects of adjuvants are an inflammation at the injection site. This effect is noticeable in farm animals such as poultry, resulting in reduction of meat quality and escalating condemnation of carcasses [125,126]. The price of vaccines is also considerably increased by adjuvants [127]. Discovering new, safe, steady, and cheap adjuvants is therefore vital to improving existing vaccines. Fossil shell flour has successfully met this need. Singh and O’Hagan [125] in their experiment using fossil shell flour as an adjuvants vaccine for Newcastle Disease Virus (NDV) for poultry, suggested that fossil shell flour could function as a prospective immunological agent for vaccines against poultry diseases. They established that using fossil shell flour as a vaccine adjuvant, causes no harmful effects to hatchability, quality of chicks, body weight, and meat quality. Although an apparent immune response was not observed when the vaccines were applied in ovo, subcutaneous boosters with NDV adjuvanted with diatoms generated NDV specific antibodies, starting at 7 d post the second booster in chickens. The effectiveness of diatoms as an adjuvant for INDV vaccines was similar to the action shown by aluminum hydroxide gel. The researchers proposed that fossil shell flour can be used as poultry adjuvant deactivated vaccines. A similar study Nazmi et al. [128] also looked at the efficacy of diatoms as adjuvants for the Ark-DPI live infectious bronchitis virus (IBV) vaccine after ocular or spray application. They observed that the addition of diatoms had no detrimental effect on the vaccine virus, hatchability, chick quality, live weight, and meat quality. However, the addition of diatoms to the vaccine did not stimulate higher IgG titers in the serum or IgA titers in tears. It also did not influence the occurrence of monocytes/macrophages in the blood and the spleen determined by flow cytometry. In addition, protection generated against IBV homologous challenges, measured by viral load in tears, respiratory signs and histopathology in tracheas, did not vary when diatoms were present in the vaccine formulation. Fossil shell flour can be postulated to be of potential as an adjuvants vaccine in Newcastle Disease Virus (NDV), although its efficacy on the Ark-DPI live infectious bronchitis virus (IBV) needs further research.

Prevention of Scours: Grazing animals frequently eat dark dirt, which contains a crystal-like type of silicon dioxide. The farmyard variety of dirt, contaminated with diseased organisms, bacteria and parasites, can lead to the death of calves and young animals. Providing stock with fresh water type of fossil shell flour that contains at least 80% pure silicon dioxide can prevent calf scours [129]. The purpose of providing fossil shell flour of free choice to the young animal is to prevent them from eating black dirt in the farmyard or elsewhere. Therefore, fossil shell flour serves as a better substitute to black dirt.

Feed additive: The nutrients and calories that are available in the diet are just as important as what the animal is fed. The digestion, health, cost-effectiveness of the diet and its related processes are also important. This is realized by maintaining a balanced intestinal process in the digestive system, and ensuring that bulk density of the feed and uniformity are within expected norms. Moreover, it is imperative to keep moisture out to avert the feed from rotting, clumping or caking.

To that effect, diatomaceous earth is a perfect animal feed additive for all livestock, with many benefits from preserving feed quality to improving livestock health and performance through better digestibility, acceptability and overall bioavailability. Diatomaceous earth will also provide cost-benefit advantages by improving mixing properties, as well as increasing the bulk density of some ingredients. The basic function of diatomaceous earth is to act as a natural preservative for the feed, absorbing moisture that may cause fungus, mold, or rot. In addition, due to its moisture reduction capacity, it reduces clumping and prevents caking of the feed. This helps to preserve feed without the need of chemicals, making it more acceptable to the animals during feeding and increasing processing and delivery efficiency.

Diatomaceous earth also helps to increase stock health, feed conversion, and ultimately, its performance. It is known to reduce internal and external parasites, bacterial infections, to control worms; its residual mineral content may also give animals a shinier coat.

However, for diatomaceous earth to work properly and efficiently as an animal feed additive, it has to be noncalcined and completely natural diatomaceous earth from fresh water. It should be organic (OMRI listed) and respect CFIA standards. Using fossil shell flour as an animal feed additive will increase return on investment by keeping the animal healthier, improving their feed conversion rates and ensuring the feed works harder and longer by preserving it naturally.

Source of natural Silica: Approximately 85% of food-grade fossil shell flour consists of silica. This significant mineral is needed by vital organs of the body for maintenance and development [97,100]. Before modern farming depleted the soil, food ingredients were the main source of natural silica. Nazmi et al. [130] and Martin [100] reported that plant-based food (natural) contains only one-third of the silica needed by mammals. With an increase in host resistance to many antihelmintics and antimicrobial drugs, coupled with health safety issues raised by the consumers of the products of animals that use the drugs, the use of fossil shell flour in livestock production is a promising substitute to these drugs [131].

In the testimony and report of [39] who experimented with FSF on his Merino sheep farm, allowing merino ewes to receive 300 g of FSF 2 weeks before lambing resulted in a good performance, and healthy, award-winning young.

Nonbeneficial effects of DE: Available information on the non-beneficial effects of DE showed that people who work with crystalline form of diatomaceous earth in large amounts such as miners, quarrymen, smelters, sandblasters, masons, and ceramic and glass manufacturers are likely to have lung problems [132,133]. Due to their very small density, Silica nanoparticles can be readily evaporated into air, and can be inhaled. Following inhalation, nanoparticles have been reported to rapidly cross the alveolar capillary barrier and penetrate into to the systemic circulation, reaching various organs [134]. Also, Cyrs et al [132] reported that after injection or skin application, nanoparticles can be distributed into the blood, causing a significant and dose-dependent platelet aggregation. Similarly, Nemmar et al. [134] observed that when rubbed on the skin, diatomaceous earth might cause wounds or loss of parts of the skin.

## 6. Conclusions

As a result of a global increase in organic farming, arising from the demand for organic livestock edible products by consumers and food safety campaign programs, diatomaceous earth has gained attention from scientists and commercial farmers. Its use has not yet been adopted in most countries, particularly in sub-Saharan Africa. There are relatively few studies investigating the potential of diatomaceous earth in livestock, especially in small stock. Besides an absence of authenticated statistical information from researchers, there is likewise a gap in knowledge concerning the application of these substances in an inorganic control program. There is a need for adequate scientific information and infrastructure to produce and continuously supply a naturally-occurring substance such as diatomaceous earth, for improving livestock production locally and internationally. There is also a need for awareness programs regarding diatomaceous earth use in the livestock industry for both farmers and consumers. Additional investigation is needed to motivate for the broader potential of diatomaceous earth as an agent of improvement in livestock production. In this context, it is assumed the diatomaceous earth will achieve a greater role in livestock production and food safety in the near future.

## Figures and Tables

**Table 1:** Composition of natural diatomite [24,25].

Chemical Content (% Weight)	Natural Diatomite
SiO_2_	82.16
Al_2_O_2_	4.89
FeO_2_	1.46
CaO	1.23
MgO	0.89
MnO_2_	0.52
KiO	0.54
NaO	0.43
TiO_2_	0.19
P_2_O_5_	0.12
Loss of Ignition	7.55

**Table 2:** Chemical composition of different sources of diatomite (%) [26,27,28,29,30].

Country/Sample	SiO_2_	Al_2_O_3_	Fe_2_O_3_	TiO_2_	Na_2_O	KO_2_	CaO	MgO	L. Others
China	82.95	5.75	1.41	0.69	0.06	0.06	0.24	0.21	7.95
Turkey	76.5	7.25	3.85	0.5	0.45	0.85	-	-	0.43
Egypt	83.6	2.24	1.07	0.17-	-	0.53_	6.17	_	4.86
Algeria	72.1	5.3	3.8	0.37	0.65	0.54	7.2	2.6	7.44
Jordan	7.25	11.42	5.81	_	7.21	0.69	1.48	0.25	0.66
Mexico	70.38	13.52	3.37	_	0.17	0.3	0.66	0.42	11.18
Guangdong Chin	90.1	_	0.3	0.4	_	_	0.5	0.2	8.5
Shengzhon, China	65	17.50	4.8	_	0.5	_	1.1	_	11.1
Morocco	62.8	9.7	11.4	_	7.3	_	_	_	8.8
China	72	7.3	4.3	_	1.8	1.2	10	1	2.4
Suizhon, China	71.35	13.26	5.5	0.08	6.7	0.11	1.94	0.15	0.91
Caldiran, Lake Van Basin, Turkey	69.7	11.5	0.65	0.65	0.08	1.4	_	_	15.3
United States	79.55	8.18	2.62	0.70	0.25	1.30	1.31	_	3.8
Kenya	84.5	3.06	1.86	0.17	1.80	0.39	1.19	0.91	6.08
Spain	88.60	0.62	0.20	0.05	3.0	0.81	0.50	0.39	5.20
Russia	79.92	6.58	3.56	0.48	1.43	0.98	0.65	0.72	4.91
Canada	89.7	3.7	1.09	0.10	0.30	0.55	0.31	0.41	3.70
Japan	86.0	5.8	1.6	0.22	0.07	0.29	0.48	0.53	4.4
Nevada	86.0	5.27	2.12	0.21	0.34	0.39	0.24	0.29	4.90
Shengzhou, China	89.6	2.5	1.8	_	1.5	_	1	_	4.5

**Table 3:** Chemical composition of natural & modified diatomite (%) [32,33].

Compound	Natural Diatomite	Modified Diatomite
SiO	63.31	56.79
Al_2_O_3_	13.42	12.15
Fe_2_O_3_	12.58	10.11
Na_2_O	0.74	2.37
CaO	0.49	0.08
Cl	<0.019	9.12
Loss on Ignition	6.54	6.73

**Table 4:** World diatomaceous earth production (in thousand metric tons) [34,35].

Country	2014	2015	2016	2017
USA	901	925	850	700
Argentina	100	55	200	200
China	420	420	420	420
Czech Republic	49	50	450	450
Denmark	95	95	440	440
France	75	75	75	75
Japan	90	100	100	100
Mexico	88	80	80	90
Peru	125	125	150	120
Russia	70	70	70	70
Spain	36	36	50	50
Turkey	85	90	60	60
Other countries	122	170	120	120

**Table 5:** Mineral constituent of fossil shell flour (FSF).

Element	Quantity
Calcium (Ca)	0.40
Sodium (Na)	0.26
Manganese (Mn)	0.0052
Iron (Fe)	0.72
Copper (Cu)	0.0019
Vanadium (V)	ppm 43.8
Sulfate Sulfur (S)	0.062
Phosphorus (as P205)	0.037
Potassium (K)	0.16
Chloride	0.074% or 740 ppm
Zinc (Zn)	0.0022
Titanium (Ti)	ppm 420
MgO (calculated from % Mg)	0.34
Strontium (Sr)	ppm 59.9
Boron (B)	0.0023
Magnesium (Mg)	0.21
% CaO (calculated from % Ca)	0.55
Aluminum (Al) %	0.65

Sources: Adebiyi et al [25].
